# Scoping review and characteristics of publicly available checklists for assessing clinical trial feasibility

**DOI:** 10.1186/s12874-022-01617-6

**Published:** 2022-05-19

**Authors:** Viktoria Gloy, Benjamin Speich, Alexandra Griessbach, Ala Taji Heravi, Alexandra Schulz, Thomas Fabbro, Christiane Pauli Magnus, Stuart McLennan, Wendy Bertram, Matthias Briel

**Affiliations:** 1 Basel Institute for Clinical Epidemiology and Biostatistics, Department of Clinical Research, University of Basel and University Hospital Basel, Spitalstrasse 12, 4031 Basel, Switzerland; 2 Centre for Statistics in Medicine, Nuffield Department of Orthopaedics, Rheumatology and Musculoskeletal Sciences, University of Oxford, Oxford, UK; 3 Clinical Trials Center, University Hospital Zurich, Zurich, Switzerland; 4 Swiss Tropical and Public Health Institute, Basel, Switzerland; 5 Clinical Trial Unit, Department of Clinical Research, University Hospital Basel, Basel, Switzerland; 6 Institute of History and Ethics in Medicine, TUM School of Medicine, Technical University of Munich, Munich, Germany; 7 Musculoskeletal Research Unit, Translational Health Sciences, Bristol Medical School University of Bristol, Bristol, UK; 8 Department of Health Research Methods, Evidence, and Impact, McMaster University, Hamilton, Canada

**Keywords:** Randomized controlled trials, Feasibility assessment, Checklist, Validation

## Abstract

**Background:**

Whether there is sufficient capacity and capability for the successful conduct and delivery of a clinical trial should be assessed by several stakeholders according to transparent and evidence-based criteria during trial planning. For this openly shared, user-tested, and validated tools are necessary. Therefore, we systematically examined the public availability and content of checklists which assess the study-level feasibility in the planning phase of clinical trials.

**Methods:**

In our scoping review we systematically searched Medline, EMBASE, and Google (last search, June 2021). We included all publicly available checklists or tools that assessed study level feasibility of clinical trials, examined their content, and checked whether they were user-tested or validated in any form. Data was analysed and synthesised using conventional content analysis.

**Results:**

A total of 10 publicly available checklists from five countries were identified. The checklists included 48 distinct items that were classified according to the following seven different domains of clinical trial feasibility: regulation, review and oversight; participant recruitment; space, material and equipment; financial resources; trial team resources; trial management; and pilot or feasibility studies. None of the available checklists appeared to be user-tested or validated.

**Conclusions:**

Although a number of publicly available checklists to assess the feasibility of clinical trials exist, their reliability and usefulness remain unclear. Openly shared, user-tested, and validated feasibility assessment tools for a better planning of clinical trials are lacking.

**Supplementary Information:**

The online version contains supplementary material available at 10.1186/s12874-022-01617-6.

## Background

Evidence-based health care relies on high quality clinical research. Randomized controlled trials (RCTs) are the method of choice to assess preventive and therapeutic interventions and are a cornerstone in the final phase of drug development and in comparative effectiveness research [1, 2]. Conducting high quality clinical trials, however, is challenging [1, 2]; requiring specialized capacities and capabilities in the areas of the clinical conduct of studies, adherence to ICH E6 Good Clinical Practice guidelines, regulatory aspects, data management, financial regulations, protection of human beings, and project management [3, 4]. Feasibility assessment during trial planning is an evaluation whether there is sufficient capacity and capability for the successful conduct and delivery of a clinical trial [5]. This trial planning process aims to ensure that the design is practical within the intended setting, the resources required for delivery are available, and recruitment targets are realistic. It is distinctly different from a feasibility study. A feasibility study “asks whether something can be done, should we proceed with it, and if so, how” [6] and it can be among other considerations part of the feasibility assessment of a trial.

If the feasibility of a clinical trial is not established before commencement of the trial, there is the risk of poor performance, insufficient recruitment, and an unacceptable high number of protocol violations [3]. Previous research indicated that one out of four RCTs are not completed as planned, primarily because of poor recruitment [7, 8], which could likely be avoided with appropriate planning [9]. The same has been found with regards to non-randomized clinical trials [10]. Poor assessment of feasibility is a known factor which adversely affects efficient trial conduct [11]. Proper feasibility assessment during the planning process can help avoid premature discontinuations of clinical trials, which constitute a considerable waste of research resources. The assessment could be done by using checklists or tools that could tell the user whether a trial is likely to be successful or needs further adjustments at the planning stage. Whereas checklists exist with the aim to improve the reporting quality of pilot- and feasibility trials [6, 12], it is currently not known, whether there are validated and publicly available checklists that could be used to assess the study level feasibility of clinical trials during their planning. Therefore, we aimed to systematically identify available checklists with a focus on whole study and not site level feasibility assessment and to examine their contents.

## Methods

A scoping review was conducted to identify feasibility checklists. Scoping reviews are used to map the existing literature, and are considered particularly suitable for complex or heterogeneous areas of research [13]. This study is reported adhering to the Preferred Reporting Items for Systematic reviews and Meta-Analyses (PRISMA) extension for Scoping Reviews [14]. No written protocol exists for this scoping review.

### Eligibility criteria

Publicly available checklists that can be used to assess study level feasibility of a clinical trial, independent of the planning stage (e.g. protocol) or source (any country, university, funding agency, clinical trial organisation), were eligible to be included in the study. We aimed to focus on study level feasibility checklists and therefore excluded checklists focusing only on site level or program level feasibility assessment (see Supplementary file and Supplementary excel file for details) [15].

### Information sources, search, and selection

We systematically searched Medline and Embase via Ovid from inception to June 2021 without any language restrictions. In addition, BS and VG independently searched the internet via Google and the homepages of relevant research stakeholder organisations with pre-specified word combinations; initially in October 2019 and updated in June 2021. The internet was searched until BS and VG felt that saturation was reached (i.e. the last 20 hits did not reveal any new relevant information). The search strategy is presented in the Supplementary information file.

Based on the eligibility criteria, VG along with either BS, AG, ATH, AS, TF, CMP, or MB screened all titles and abstracts of references found through the literature search for potentially eligible publications. Full texts of potentially eligible checklists were then independently screened by VG and either BS, ATH, AG, or MB. In case of disagreement, consensus was reached by discussion.

### Data extraction

Of the included checklists, VG extracted information on source, country name, institution type, intended users, any description of user testing or validation, whether the checklist is provided with any instructions on how it has to be applied or how the result should be interpreted. All extractions were cross-checked by BS, ATH, or AG.

### Analysis

Using the included checklist documents, VG performed conventional content analysis [16], focusing on items or themes common across checklists as well as those unique to individual policies. VG read and coded all checklists, with initial themes being identified inductively using a process of open coding (i.e., no specific preconceived codes were identified or used; rather, codes emerged directly from the data). A coding framework was developed by a progressive process of classifying, comparing and refining text passages to create categories. The final coding framework was checked by the other co-authors to ensure consistency and validity. The results of the analysis were summarized in tables. One presenting characteristics of the included checklists and the other presenting the identified items that may determine study level feasibility. In terms of critical appraisal of individual checklists, we checked whether they were user-tested or validated. The raw data can be found in a Supplementary excel file.

## Results

### Selection of sources

**Fig. 1:**
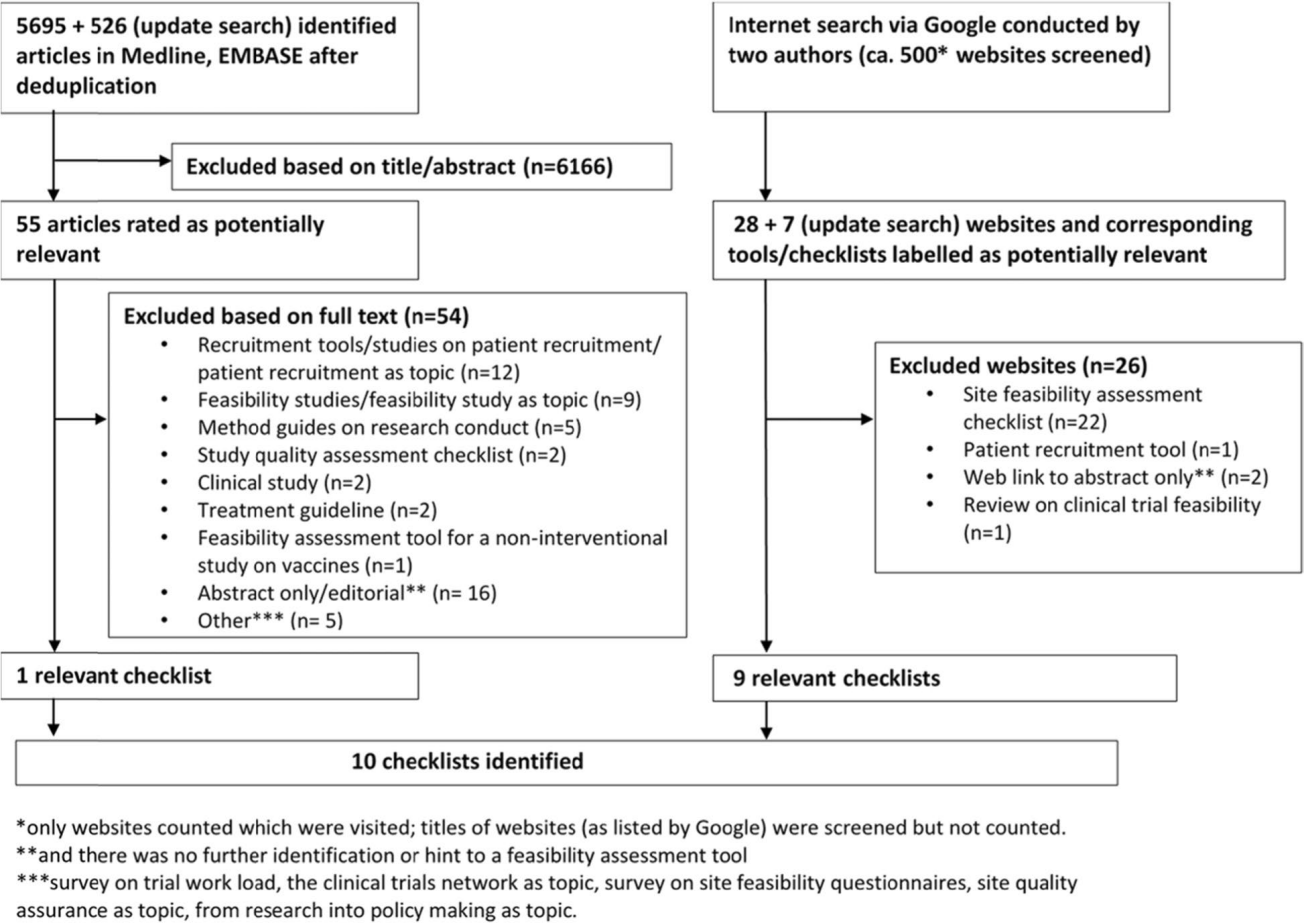
Results of the information search

The literature search identified 6221 references of which one was included in the final analysis, and the internet search identified 35 potential checklists of which nine were included (Fig. 1). Thus, in total we identified 10 publicly available checklists to determine study level feasibility of clinical trials.

### Characteristics of included checklists

Out of the 10 included checklists, 5 were issued by universities [5, 17–20], 3 by health care facilities [21–23], and 2 by national research organisations [24, 25] (Table 1). The checklists were from a total of 5 different countries, nine checklists were published in English and one in German. Intended users of the checklists were funders and investigators [24], health care facilities and investigators [5, 25], funders [17], investigator only [18, 19], investigator and department chair or designee [20] or investigator and research facilitator [21]. In one case, a university department required investigators to submit the filled checklist to their institutional review board before approval of a new trial [20].

**Table 1:** Characteristics of the included checklists

Source	Country (language)	Institution type Intended user	Is indicated based on which information the assessment has to be done? Is indicated whether the checklist is implemented and used on a regular basis?*	Explanations how to fill out the checklist? User testing or validation mentioned? Evidence base mentioned (e.g. expert consensus)?	Number of items (according to our coding) included in the checklist
National Institute for Health Research, Association of Medical Research Charities (AMRC) and NIHR Medicines for Children Research Network (MCRN) [24]	United Kingdom (English)	National research Organization Funder and investigator	Yes (grant proposal) No	No/no/no	27
Clinical research centre, ministry of health Malaysia, Institute for clinical research [25]**	Malaysia (English)	National research Organization Health care facility and investigator	No No	No/no/no	23
University of Calgary [17]	Canada (English)	University Funder	No No	No/no/no	21
The University of North Carolina, Office of Clinical Trials [18]	USA (English)	University Investigator	No No	No/no/no	17
Clinical trial unit, University Hospital Basel [19]	Switzerland (German)	University Investigator	No No	No/no/no	15
University of Bristol, Bristol Medical School [5]	United Kingdom (English)	University Health care facility and investigator	No No	No/no/no	26
University of Wisconsin-Madison, Institute for Clinical and Translational Research, [20]	USA (English)	University Investigator and Department Chair or Designee	Yes (protocol) Yes	No/no/no	13
Kings College Hospital, National Health Service foundation trust [21]	United Kingdom (English)	Health care facility Investigator and research facilitator	No No	No/no/no	28
Health First [22]	USA (English)	Health care facility Health care facility and investigator	Yes (protocol, informed consent form, other facility specific forms etc.) No	No/no/no	7
University of Florida, Health Cancer Center feasibility group Clinical Research Office [23]	USA (English)	Health care facility Investigator	Yes (protocol) No	No/no/no	12

Only four checklists indicated the information required for a feasibility assessment: trial protocol only [20, 23], informed consent form, and other facility specific forms [22], or grant proposal [24]. None of the included checklists provided information on whether they were user tested or validated. With the exception of one checklist [23], they did not provide any instructions on how to fill out the checklist or how to interpret the results of the assessment. In addition, the underlying evidence-base for the development of the identified checklists (e.g., whether the checklist items were based on expert consensus) was missing for all checklists.

### Feasibility assessment items

A total of 48 distinct items in relation to assessing the feasibility of a clinical trial were identified (Table 2). These items were further categorised into 7 domains: regulation, review and oversight; participant recruitment; space, material and equipment; financial resources; trial team resources; trial management; and pilot or feasibility studies.

**Table 2:** List of identified items for study-level feasibility assessment of clinical trials

**Domain**	**Items**	**Number of checklists, total = 10** [References]
1. Regulation, review and oversight	The clinical trial is compliant with local regulations	7 [5, 17–19, 21, 23, 25]
	The study protocol has been (independently) peer reviewed	2 [21, 23]
	Safety aspects are accommodated	3 [20, 21, 23]
2. Participant recruitment	The target population is available	8 [5, 17, 18, 20–23, 25]
	Competing trials are known	7 [5, 17, 18, 21, 23–25]
	A recruitment rate is estimated	6 [18, 19, 21–24]
	Factors that hinder/have an impact on recruitment are known	5 [5, 21, 23–25]
	The target sample size to recruit is known	5 [5, 18, 19, 21, 22]
	The study is interesting to others (e.g. physicians, co-investigators)	5 [5, 18, 20, 21, 24]
	Strategies and resources that are needed for recruitment are known	4 [5, 17, 21, 24]
	Routine data sources corroborate estimated recruitment rate or can facilitate recruitment	4 [5, 17, 23, 24]
	Eligibility criteria are clear and realistic	3 [17, 18, 25]
	Other sites are available, if necessary	3 [17, 24, 25]
	Organisations and groups, relevant to recruitment, are known	2 [5, 24]
	How the target sample size was calculated is known	2 [5, 21]
	The necessary number of sites is known	2 [17, 19]
	The estimated recruitment rate(s) is/are reasonable	1 [23]
3. Space, material and equipment	Access to professional support and required facilities is available	8 [5, 17–19, 21, 22, 24, 25]
	Equipment is appropriate and sufficient	8 [5, 17–20, 22, 24, 25]
	Working space is appropriate and sufficient to conduct the study	6 [5, 17, 19, 21, 24, 25]
	Study drug and comparator are available	7 [17–21, 24, 25]
	Storage room for study material is appropriate and sufficient	5 [5, 17, 19, 21, 25]
	Secure storage room for study or patient documents/ recorded data is sufficient	6 [5, 17, 19–21, 25]
	Access to relevant electronic systems are available	5 [5, 18–21]
4. Financial resources	The budget is adequate	6 [17, 18, 20, 21, 23, 25]
	Excess costs at sites are accommodated	3 [5, 21, 24]
	The budget for recruitment and follow-up visits is adequate	1 [19]
5. Trial team resources	Adequate staffing is identified and available within the trial period	8 [5, 17–21, 24, 25]
	Investigator / study team has time for study visits	5 [5, 17, 20, 21, 24, 25]
	Investigator has time to complete the study	4 [5, 21, 24, 25]
	Training for staff is available	4 [5, 18, 21, 24]
	Investigator has appropriate experience	3 [21, 24, 25]
	Investigator has time to supervise the trial team	4 [17, 20, 24, 25]
	Investigator has time to check the data	2 [17, 25]
	Investigator has time to interact with the sponsor	2 [17, 25]
	Work out of hours is accommodated	2 [5, 21]
	Investigator has capacity to recruit the patients	1 [25]
6. Trial management	Current standard of practice at trial site is compatible with trial protocol	8 [5, 17, 18, 20–24]
	The study schedule is reasonable	4 [17, 18, 22, 25]
	Specific patient related aspects are accommodated (e.g. children)	3 [21, 23, 24]
	The methods for site selection are known	3 [5, 20, 24]
	Project management considerations were made	2 [5, 19]
	Special vendor requirements are known	2 [18, 21]
	On-site management is available	1[19]
	Clinical care for trial participants is coordinated and managed	1 [24]
	Assessment of outcomes is accommodated at sites	1 [24]
7. Pilot or feasibility studies	A pilot study was conducted	2 [5, 24]
	All sites were included in the feasibility studies	1 [24]

The number of distinct items according to our classification ranged in checklists from 7 to 28 with a median of 21 items (Table 2). There were four checklists that included about half of all identified 48 items and the other six checklists less than that. The domain with the highest number of identified distinct items was participant recruitment (14 items), followed by trial team resources (11 items). All other domains contained nine or less items.

The most frequently mentioned items (mentioned by 8/10 checklists) across all domains were “The target population is available “ (domain: participant recruitment), "Access to professional support and required facilities is available" and “Equipment is appropriate and sufficient “ (domain: Space, equipment and material), "Current standard practice at trial site(s) is compatible with trial protocol" (domain: Trial management), and “Adequate staffing is identified and available within the trial period” (domain: Trial team resources). Also frequent (mentioned by 7/10 checklist) were the items: “The clinical trial is compliant with local regulations”, “Competing trials are known”, “Study drug and comparator are available” and “Adequate staffing is identified and available within the trial period” (Table 2).

## Discussion

This scoping review found ten checklists issued by universities, national research organizations, or health care facilities that are publicly available to assess study level feasibility of clinical trials. We identified 48 distinct items for trial feasibility assessment. The most frequently mentioned individual items were “The target population is available “, "Access to professional support and required facilities is available", “Equipment is appropriate and sufficient “, "Current standard practice at trial site(s) is compatible with trial protocol" and “Adequate staffing is identified and available within the trial period”. The number of items differed considerably across feasibility checklists. Only four of the ten checklists contained about half of the identified 48 items, the other five checklists less than that. For only four of the identified checklists the documentary basis (e.g. trial protocol) for the assessment was specified, and for none of the checklists the choice of items was justified or the way of compiling items explained. None of the available checklists appeared to be user-tested or validated. Thus, the validity, practicability of available trial feasibility checklists, and whether or not the implementation of such checklists indeed leads to more successful trial conduct appears uncertain. No single checklist is likely to cover all the items required to assess feasibility for every trial and is reliant on the user completing the checklist as intended [26]. Furthermore, checking for feasibility during trial planning has to be seen in the context of a comprehensive framework of clinical research that covers all stages of a clinical trial, i.e. concept, planning and feasibility, conduct, analysis and interpretation, and reporting and knowledge translation [27]. Trial success may also depend on these other phases. Thus, equivalent tools are conceivable for the other phases, too. For example, the implementation of a risk- based monitoring tool during trial conduct.

### Comparison to other literature

Although there is substantial literature on feasibility studies, reporting guidelines, and since 2015 even an online journal fully dedicated to pilot and feasibility studies exists [6, 12, 28], the actual assessment whether there is sufficient capacity and capability for the successful conduct and delivery of a clinical trial seems to be a neglected topic in the literature. We only found a single article of a publicly available feasibility checklist with our systematic literature search [5]. There are viewpoints, commentaries, or perspectives articles discussing different aspects of clinical trial feasibility without providing a practical checklist or describing scientific work for a systematic tool development [3, 11, 15]. The here mentioned key factors for trial feasibility assessment largely overlap with the domains from our content analysis. Butryn et al., for instance, considered optimal resource allocation, operational efficiency, financial viability, and enrolment success as essential components for trial feasibility; and the success of each component is best achieved through close collaboration between the principal investigator, the research team, information technology specialists, and ancillary departments (e.g. radiology) [3]. As a reaction to another prematurely discontinued RCT due to poor recruitment, an editorial by Maas raised the overdue question about criteria for pre-study feasibility assessment and suggested that clinical trial registries such as clinicaltrials.gov should consider requiring information about trial feasibility assessments [29]. Given the high prevalence of premature trial discontinuations due to recruitment or organisational problems [7, 8], and the associated huge amount of wasted resources, it is surprising that the clinical trial community has not yet adequately responded to the obvious need for more effective trial feasibility assessment.

### Limitations

Our scoping review has the following limitations: First, we might have missed available trial feasibility checklists despite our comprehensive search strategy including an internet search in addition to a literature search of two large electronic databases [30]. We chose this approach, because we assumed that we had to rely on websites and online publications of research institutions. Inherent risks of searching the internet are selection bias (bubble effect) and the issue of limited reproducibility due to the non-transparent and non-consistent search algorithm by Google.com [31]. Second, we focused only on publicly available checklists. Searching for unpublished checklists or tools would have required a different approach (e.g. survey of clinical trial stakeholders). However, in our opinion this is a minor limitation as we aimed to provide an overview of publicly available tools that can also be accessed by any stakeholder. Third, we could not assess the quality of the identified checklists since we did not find any information on how they were developed. A detailed description of advantages and disadvantages of the different checklists would require comprehensive user testing, ideally using a sample of RCTs that are currently in the planning phase. Fourth, the provided overview of suggested feasibility assessment items is not a recommendation for how an ideal checklist should look like (e.g. not all items might be relevant to trial success or some items may be missing) and is not ready to implement – it is rather a first step for systematic and transparent tool development (see Future directions).

These limitations, however, are hardly relevant for our conclusion that user-tested and validated clinical trial feasibility assessment checklists or tools are lacking. We think that our search allowed us to identify the available checklists that an investigator would find who probably conducts less-extensive searches of the internet or literature databases.

### Future directions

Our overview of suggested items for trial feasibility assessment may be used as a starting point for the systematic and transparent development of a reliable, valid, and user-friendly feasibility assessment tool involving relevant stakeholders such as trial investigators, trial support organizations, research ethics committees, and funding agencies. A large international group of stakeholders could first examine whether there are any missing items, more or less important items (grading the importance of items) and bring forward feasibility checklists or tools that are not publicly available. A resultant item list could then undergo a consensus process across stakeholders using the Delphi technique to determine which items need to be considered in an effective trial feasibility checklist and how assessment results should be applied. Subsequently, empirical user testing and validation work is important. A similar tool development process has recently been successfully completed for an assessment of subgroup effect credibility [32]. Finally, evidence needed to be generated (e.g. a cohort of trials either randomised to using a feasibility checklist or not) in order to investigate whether the implementation of a feasibility checklist indeed leads to more successful trial conduct (e.g. measured by enrolment success). Furthermore, empirical research needs to establish how trial success is associated with individual items that appear relevant for study level feasibility. It might well be that some of these items are gatekeepers and, thus, more important than others for trial success (e.g. whether or not a pilot trial was conducted).

## Conclusions

This scoping review identified ten publicly available checklists to assess the feasibility of RCTs. None of the available checklists appeared to be user-tested or validated. We extracted 48 distinct items for trial feasibility assessment that can be grouped into seven categories. Our results only describe the currently available checklists and suggested assessment items, and we make no recommendations at this stage of the project on how to assess the feasibility of a clinical trial. Instead, we provide the evidence-base for the transparent development of an improved checklist or tool for trial feasibility assessment, including user testing and validation and encourage relevant stakeholders, e.g. university hospitals, research ethics committees, funding agencies to get involved.

## Supplementary Information

**Additional file 1.**

**Additional file 2.**

## Data Availability

Details on the information search and the Preferred Reporting Items for Systematic reviews and Meta-Analyses extension for Scoping Reviews (PRISMA-ScR) Checklist can be found in a Supplementary information file. The raw data can be found in a Supplementary excel file.
