# Supplementary material for: Scoping review and characteristics of publicly available checklists for assessing clinical trial feasibility
Source: BMC Med Res Methodol. 2022 May 19;22:142. doi: 10.1186/s12874-022-01617-6 (PMC9118562; doi:10.1186/s12874-022-01617-6)
Supplement: Supplementary file 1 — Additional file 1. [file 12874_2022_1617_MOESM1_ESM.docx]

# **Appendix**

## Literature search

#### Medline search strategy

Ovid MEDLINE(R) ALL 1946 to September 19, 2019, update on the 16^th^ of June 2021 (limit search to dt=20190901-20210616)

((feasibility OR feasible OR practical OR practicability OR operational) **adj10** (test OR test? OR testing OR check OR check? OR checking OR assessment? OR assessing OR assess OR valuation? OR valuating OR valuate OR appraisal OR appraising OR appraise OR evaluation? OR evaluating OR evaluate OR indicator? OR tool? OR template? OR framework? OR guideline OR review OR instrument? OR recommendation? OR checklist OR component? OR item? OR aspect? OR question?)).ab,ti.

**OR**

(Checklist/ OR Practice Guidelines as Topic/) AND (feasibility OR feasible OR practical OR practicability).ab,ti.

AND

Controlled Clinical Trials as Topic/ OR clinical studies as topic/ OR Randomized Controlled Trials as Topic/ OR clinical trials as topic/ OR clinical trial? as Topic.mp. OR randomised controlled trial? as Topic.mp. OR research design/mt, st OR Biomedical Research/mt, st

#### Embase search strategy

Embase 1974 to 2019 September 19, update on the 16^th^ of June 2021 (limit search to dd=20190901-20210616)

((feasibility OR feasible OR practical OR practicability OR operational) adj10 (test OR test? OR testing OR check OR check? OR checking OR assessment? OR assessing OR assess OR valuation? OR valuating OR valuate OR appraisal OR appraising OR appraise OR evaluation? OR evaluating OR evaluate OR indicator? OR tool? OR template? OR framework? OR guideline OR review OR instrument? OR recommendation? OR checklist OR component? OR item? OR aspect? OR question?)). ab,ti.

**OR**

(Practice guideline/ OR checklist/) AND (feasibility OR feasible OR practical OR practicability) .ab,ti.

AND

"controlled clinical trial (topic)"/ OR "randomized controlled trial (topic)"/ OR "clinical trial (topic)"/ OR clinical trial? as Topic.mp. OR randomised controlled trial? as Topic.mp.

Legend: .mp. = keyword; / = mesh term; mt = methods; st = standards

## Internet search

(Conducted between 21. and 24. of October 2019 and updated between 17th and 21st of June 2021)

#### Search on webpages of relevant research stakeholder organizations

Webpages will be searched in the respective country specific languages.

**National Clinical Research Associations:**

1. Swiss Group for Clinical Cancer Research (SAKK) Switzerland
2. Schweizerische Akademie der Medizinischen Wissenschaften (SAMW) Switzerland
3. Clinical Research Association of Canada (expert) Canada
4. Institut National de la santé et de la recherche médicale (INSERM): Reseau des CIC (expert) France
5. Arbeitsgemeinschaft der Wissenschaftlichen Medizinischen Fachgemeinschaften (AWMF) (expert) Germany
6. Deutsches Netzwerk Evidenz Basierte Medizin (EBM) (expert) Germany
7. Netherlands Institute for Health Sciences Netherlands
8. Japan Society of Clinical Trials and Research (expert) Japan
9. UCSF Clinical Research HUB USA

**International Clinical Research Associations:**

1. European Clinical Research Infrastructure Network

**National Academic Research Organization Networks:**

1. Swiss Clinical Trial Organisation (SCTO) Switzerland
2. The Association of Canadian Academic Healthcare Organisations Canada
3. Koordinierungszentrum für Klinische Studien (KKS Netzwerk) Germany
4. AcademyHealth USA

**Supranational and National Funding Agencies / Programs**

1. European Science Foundation (ESF) EU
2. National Health and Medical Research Council Australia
3. Canadian Institute for Health Research (CIHR) Canada
4. Programme Hospitalier de Recherche Clinique France
5. Deutsche Forschungsgemeinschaft (DFG) - Bundesministerium für Bildung und Forschung (BMBF) Germany
6. Robert-Koch-Institut Germany
7. Agenzia Italiana del Farmaco (AIFA) Fund Italy
8. Ministry of Health (Research and Development Program) Italy
9. Japan Society for the Promotion of Science (JSPS) Japan
10. Japan Science and Technology Agency (JST) Japan
11. The Research Council Norway
12. Instituto de Salud Carlos III Spain
13. Vetenskapsradet (Swedish Research Council) Sweden
14. Schweizer Nationalfonds Switzerland
15. Medical Research Council UK
16. Patient Centered Outcome Research Institute (PCORI) USA
17. NIH/NIH-The Common Fund USA
18. Agency for Healthcare Research & Quality (AHRQ) (expert) USA

#### Google web search

Additional word combinations will be added independently by the authors. Whereas we will do an extensive search in English, we will do a restricted search in German and French, as well.

1. (feasibility OR feasible OR practical OR practicability) AND (tool OR checklist) AND (clinical trial OR clinical study OR RCT OR randomized OR randomised)
2. Feasibility random* trial tool
3. Feasibility random* trial checklist
4. Practicability random* trial tool
5. Practicability random* trial checklist
6. Feasibility clinical research tool
7. Feasibility clinical research checklist
8. Evaluation feasibility AND (clinical trial OR clinical research)
9. Evaluation practicability AND (clinical trial OR clinical research)
10. Appraisal AND (feasibility OR practicability) AND (clinical trial OR clinical research)
11. Template AND (feasibility OR practicability assessment) AND (clinical trial OR clinical research)
12. Framework AND (feasibility OR practicability assessment) AND (clinical trial OR clinical research)
13. Guideline (feasibility OR practicability assessment) AND (clinical trial OR clinical research)
14. Instrument (feasibility OR practicability assessment) AND (clinical trial OR clinical research)
15. Recommendation (feasibility OR practicability assessment) AND (clinical trial OR clinical research)

In the following, country names are only given for orientation, need not to be used for the search. The slash indicates that the search should be done with the translated word as well.

1. Feasibility assessment/ Machbarkeit Swiss Group for Clinical Cancer Research (SAKK) Switzerland
2. Feasibility assessment/Machbarkeit Schweizerische Akademie der Medizinischen Wissenschaften (SAMW) Switzerland
3. Feasibility assessment Clinical Research Association of Canada (expert) Canada
4. Feasibility assessment/ faisabilité Institut National de la santé et de la recherche médicale (INSERM): Réseau des CIC (expert) France
5. Feasibility assessment/Machbarkeit Arbeitsgemeinschaft der Wissenschaftlichen Medizinischen Fachgemeinschaften (AWMF) (expert) Germany
6. Feasibility assessment Deutsches Netzwerk Evidenz Basierte Medizin (EBM) (expert) Germany
7. Feasibility assessment Japan Society of Clinical Trials and Research (expert) Japan
8. Feasibility assessment UCSF Clinical Research HUB USA
9. Feasibility assessment/Machbarkeit Swiss Clinical Trial Organisation (SCTO) Switzerland
10. Feasibility assessment The Association of Canadian Academic Healthcare Organisations Canada
11. Feasibility assessment/Machbarkeit Koordinierungszentrum für Klinische Studien (KKS Netzwerk) Germany
12. Feasibility assessment AcademyHealth USA
13. Feasibility assessment/ faisabilité European Science Foundation (ESF) EU
14. Feasibility assessment/Machbarkeit National Health and Medical Research Council Australia
15. Feasibility assessment Canadian Institute for Health Research (CIHR) Canada
16. Feasibility assessment/ faisabilité Programme Hospitalier de Recherche Clinique France
17. Feasibility assessment/Machbarkeit Deutsche Forschungsgemeinschaft (DFG) - Bundesministerium für Bildung und Forschung (BMBF) Germany
18. Feasibility assessment/Machbarkeit Robert-Koch-Institut Germany
19. Feasibility assessment Agenzia Italiana del Farmaco (AIFA) Fund Italy
20. Feasibility assessment Ministry of Health (Research and Development Program) Italy
21. Feasibility assessment Japan Society for the Promotion of Science (JSPS) Japan
22. Feasibility assessment Japan Science and Technology Agency (JST) Japan
23. Feasibility assessment The Research Council Norway
24. Feasibility assessment Instituto de Salud Carlos III Spain
25. Feasibility assessment Vetenskapsradet (Swedish Research Council) Sweden
26. Feasibility assessment/ Machbarkeit Schweizer Nationalfonds Switzerland
27. Feasibility assessment Medical Research Council UK
28. Feasibility assessment Patient Centered Outcome Research Institute (PCORI) USA
29. Feasibility assessment NIH/NIH-The Common Fund USA
30. Feasibility assessment Agency for Healthcare Research & Quality (AHRQ) (expert) USA
31. faisabilité Clinique
32. Machbarkeit klinische Forschung

**APPENDIX TABLE: List of excluded checklists because the identified purpose was site level feasibility assessment**

| SOURCE of INFORMATION | ID | REASON FOR EXCLUSION |
| --- | --- | --- |
|  |  |  |
| <https://hub.ucsf.edu/sites/g/files/tkssra261/f/Clinical%20Trial%20Feasibility%20Checklist.pdf> | 16 | ex (site feasibility assessment) |
| <https://accrualnet.cancer.gov/sites/accrualnet.cancer.gov/files/Icahn%20School%20of%20Medicine%20at%20Mount%20Sinai.pdf> | 7 | ex (site feasibility assessment) |
| https://www.uab.edu/ccts/images/Comprehensive__FEASABILITY_FORM_1-21-15.pdf | 14 | ex (site feasibility assessment) |
| https://hub.ucsf.edu/sites/g/files/tkssra261/f/FeasibilityChecklist_APRIL20132013.xls | 10 | ex (site feasibility assessment) |
| <https://www.rochester.edu/ohsp/documents/quality/word/documentationNTF/ClinicalTrialFeasibilityChecklist.docx> | 12 | ex (site feasibility assessment) |
| https://www.researchgo.ucla.edu/sites/default/files/sites/default/FeasibilityChecklist_MAY2016.xls+&cd=10&hl=en&ct=clnk&gl=uk | 15 | ex (site feasibility assessment) |
| <https://irb.upenn.edu/announcements/clinical-research-resource-feasibility-assessment-tool> | 4 | ex (site feasibility assessment) |
| https://www.nwh.org/media/file/Research%20Investigator%20Forms/Clinical%20Trial%20Feasibility%20Guide.pdf | 9 | ex (site feasibility assessment) |
| https://globalhealthtrials.tghn.org/site_media/media/medialibrary/2015/08/Site_Assessment_and_Feasibility_Questionnaire.doc | 6a | ex (site feasibility assessment) |
| <https://globalhealthtrials.tghn.org/site_media/media/medialibrary/2015/08/Protocol_Feasibility_Assessments_SOP.docx> | 6b | ex (site feasibility assessment) |
| <https://www.fraserhealth.ca/-/media/Project/FraserHealth/FraserHealth/Health-Professionals/Research-and-Evaluation-Services/Clinical-Research-and-Start-up-Toolkit/Clinical-research-study-completion/20170601-site-feasibility-template.doc> | 5 | ex (site feasibility assessment) |
| <https://irb.research.chop.edu/study-planning-feasibility-assessment> | 3 | ex (site feasibility assessment) |
| https://studylibfr.com/doc/143378/liste-de-v%C3%A9rification-de-la-faisabilit%C3%A9-d-un-essai-clinique | 20 | ex (site feasibility assessment) |
| <http://www.health.act.gov.au/sites/default/files/2018-10/Canberra%20Hospital%20Feasibility%20Assessment%20Registry.xlsx> | E | ex (site feasibility assessment) |
| <http://palliativecare.walescancerresearchcentre.com/uploads/Toolkit/Feasibility_checklist_final_20-09-13_updated_LU_AW.pdf> | F | ex (site feasibility assessment) |
| <https://www.uth.edu/ctrc/documents/ChecklistFeasibilityQuestionnaire.doc> | G | ex (site feasibility assessment) |
| <https://irb.research.chop.edu/study-planning-feasibility-assessment> | **H** | ex (site feasibility assessment) |
| <https://www.nwh.org/media/file/Research%20Investigator%20Forms/Clinical%20Trial%20Feasibility%20Guide.pdf> | I | ex (site feasibility assessment) |
| <https://genesisresearchservices.com/clinical-trial-feasibility-and-capacity-planning-tool/> | J | ex (site feasibility assessment) |
| <https://www.ohsu.edu/sites/default/files/2019-12/2019-12-12%20OCTRI%20RF%20Feasibility%20Handout%20Final.pdf> | K | ex (site feasibility assessment) |
| <https://www.iths.org/wp-content/uploads/ResearchStudyFeasibilityTool_V12017Feb6.pdf> | M | ex (site feasibility assessment) |
| <https://compass.ucsd.edu/sites/default/files/sites/default/FeasibilityChecklist_MAY2016.xls> | N | ex (site feasibility assessment) |

**Preferred Reporting Items for Systematic reviews and Meta-Analyses extension for Scoping Reviews (PRISMA-ScR) Checklist**

| **SECTION** | **ITEM** | **PRISMA-ScR CHECKLIST ITEM** | **REPORTED ON PAGE #** |
| --- | --- | --- | --- |
| **TITLE** | | | |
| Title | 1 | Identify the report as a scoping review. | 1 |
| **ABSTRACT** | | | |
| Structured summary | 2 | Provide a structured summary that includes (as applicable): background, objectives, eligibility criteria, sources of evidence, charting methods, results, and conclusions that relate to the review questions and objectives. | 3 |
| **INTRODUCTION** | | | |
| Rationale | 3 | Describe the rationale for the review in the context of what is already known. Explain why the review questions/objectives lend themselves to a scoping review approach. | 4 |
| Objectives | 4 | Provide an explicit statement of the questions and objectives being addressed with reference to their key elements (e.g., population or participants, concepts, and context) or other relevant key elements used to conceptualize the review questions and/or objectives. | 4 |
| **METHODS** | | | |
| Protocol and registration | 5 | Indicate whether a review protocol exists; state if and where it can be accessed (e.g., a Web address); and if available, provide registration information, including the registration number. | 5 |
| Eligibility criteria | 6 | Specify characteristics of the sources of evidence used as eligibility criteria (e.g., years considered, language, and publication status), and provide a rationale. | 5 |
| Information sources* | 7 | Describe all information sources in the search (e.g., databases with dates of coverage and contact with authors to identify additional sources), as well as the date the most recent search was executed. | 5 |
| Search | 8 | Present the full electronic search strategy for at least 1 database, including any limits used, such that it could be repeated. | 5 and Appendix |
| Selection of sources of evidence† | 9 | State the process for selecting sources of evidence (i.e., screening and eligibility) included in the scoping review. | 5,6 |
| Data charting process‡ | 10 | Describe the methods of charting data from the included sources of evidence (e.g., calibrated forms or forms that have been tested by the team before their use, and whether data charting was done independently or in duplicate) and any processes for obtaining and confirming data from investigators. | 5,6 |
| Data items | 11 | List and define all variables for which data were sought and any assumptions and simplifications made. | 5,6 |
| Critical appraisal of individual sources of evidence§ | 12 | If done, provide a rationale for conducting a critical appraisal of included sources of evidence; describe the methods used and how this information was used in any data synthesis (if appropriate). | Not done |
| Synthesis of results | 13 | Describe the methods of handling and summarizing the data that were charted. | 6 |
|  | | | |
| Selection of sources of evidence | 14 | Give numbers of sources of evidence screened, assessed for eligibility, and included in the review, with reasons for exclusions at each stage, ideally using a flow diagram. | 7 and Figure 1 |
| Characteristics of sources of evidence | 15 | For each source of evidence, present characteristics for which data were charted and provide the citations. | Table 1 , p.7,8 |
| Critical appraisal within sources of evidence | 16 | If done, present data on critical appraisal of included sources of evidence (see item 12). | Not done |
| Results of individual sources of evidence | 17 | For each included source of evidence, present the relevant data that were charted that relate to the review questions and objectives. | 8-12, Table 2 |
| Synthesis of results | 18 | Summarize and/or present the charting results as they relate to the review questions and objectives. | 8-12, Table 2 |
|  | | | |
| Summary of evidence | 19 | Summarize the main results (including an overview of concepts, themes, and types of evidence available), link to the review questions and objectives, and consider the relevance to key groups. | 12 |
| Limitations | 20 | Discuss the limitations of the scoping review process. | 13 |
| Conclusions | 21 | Provide a general interpretation of the results with respect to the review questions and objectives, as well as potential implications and/or next steps. | 15 |
|  | | | |
| Funding | 22 | Describe sources of funding for the included sources of evidence, as well as sources of funding for the scoping review. Describe the role of the funders of the scoping review. | 16 |

*From:* Tricco AC, Lillie E, Zarin W, O'Brien KK, Colquhoun H, Levac D, et al. PRISMA Extension for Scoping Reviews (PRISMA-ScR): Checklist and Explanation. Ann Intern Med. ;169:467–473. doi: 10.7326/M18-0850
